# Understanding spectator loyalty in the Chinese super league: the roles of satisfaction, trust, and team identification

**DOI:** 10.3389/fpsyg.2026.1776215

**Published:** 2026-03-27

**Authors:** Fei Liu, Sisi Wu, Jingyin Zhou, Mu Fan, Fengqin Tian

**Affiliations:** 1 Football School, Guangzhou Sport University, Guangzhou, China; 2 Department of Sport Science, Zhejiang University, Hangzhou, China; 3 Sports Management School, Guangdong Vocational Institute of Sport, Guangzhou, China

**Keywords:** mediation effect, professional football league, spectator loyalty, spectator satisfaction, spectator trust, team identification

## Abstract

**Purpose:**

This study aims to examine the distinct roles and sequential order of satisfaction, trust, and team identification in shaping loyalty among spectators of the Chinese Super League (CSL).

**Methods:**

Measurement items were developed based on prior literature and expert consultation, followed by a pilot study to refine the scales. Data were collected through a survey of spectators attending CSL matches, yielding 278 valid responses. Confirmatory factor analysis and mediation analyses were conducted to test the proposed structural relationships among the variables.

**Results:**

Spectator satisfaction did not exhibit a significant direct effect on loyalty (path coefficient = 0.076, *p* > 0.05), nor did trust (path coefficient = 0.037, *p* > 0.05). In contrast, team identification had a significant direct effect on loyalty (path coefficient = 0.759, *p* < 0.05). Further analysis revealed three meaningful indirect pathways underlying loyalty formation: Satisfaction→Team Identification→Loyalty (effect size = 0.242), Team Identification→Satisfaction→Loyalty (effect size = 0.071), and Satisfaction→Trust→Team Identification→Loyalty (effect size = 0.034).

**Implications:**

While improving game-related and service-related satisfaction and fostering trust remain important, CSL authorities and clubs should place greater strategic emphasis on enhancing team identification. Cultivating a strong sense of belonging and psychological attachment to the team is critical for sustaining a stable, loyal fan base.

## 1 Introduction

High-level professional football leagues exert strong driving forces on national economic and social development. For example, during the 2019/20 season, the Premier League recorded the average stadium attendance exceeding 40,000 for the first time, with a stadium utilization rate of 98.7%. The league generated approximately £3.6 billion in tax revenue and created 94,000 jobs in the United Kingdom (Premier League, 2024). The Chinese Super League (CSL), established in 2004 with the Premier League as its model, is the highest tier of professional football league in China. However, the league’s development has experienced considerable fluctuations. It currently faces several severe challenges: relatively low brand value, persistent financial losses among clubs, and an average attendance that lags behind major European leagues. The National Development and Reform Commission issued the Medium and Long-Term Development Plan for Chinese Football (2016–2050), which identified as a key reform objective the need to “expand the influence of professional leagues, enhance the brand value of the CSL, and raise average attendance to a world-leading level.”

Spectators are not only participants and consumers of sporting events but also evaluators. Their loyalty influences club revenue, league brand value, sponsorship investment, and media impact (Antonakakis, 2025). With the globalization of sports, major European leagues, such as the Premier League and La Liga, are increasingly competing for the attention and consumption resources of Chinese spectators through global media distribution and international brand strategies. Meanwhile, the rapid development of digital media and mobile internet has provided diverse entertainment options, which often divert spectators’ attention away from sporting events. Therefore, how CSL can effectively attract more spectators and strengthen its loyalty in a competitive environment has become a central issue for its sustainable development.

Spectator engagement in professional sport is an ongoing process of relational interaction. Beyond economic exchange through event attendance and consumption, spectators develop deeper connections with clubs through emotional investment and identity construction. The relationship quality between spectators and sports teams or organizations is critical for fostering spectator loyalty (Lee, M. A. et al., 2020; Lee, H. W. et al., 2020). In sports contexts, satisfaction, trust, and team identification are widely recognized as core components of relationship quality (Nolan et al., 2024). Existing research has extensively examined the individual relationships between each of these constructs and loyalty. Few studies, however, have combined these constructs into a unified framework to assess their joint effects. As a result, there is no consensus on the sequence order by which these constructs influence loyalty. This gap is clear in emerging football markets like China, where empirical research on these variables remains limited.

Therefore, this study focuses on spectators of the CSL to systematically examine the distinct roles and sequential relationships among spectator satisfaction, trust, and team identification in shaping loyalty. By clarifying the psychological pathways underlying the formation of spectator loyalty, this study extends the literature on Chinese sports consumer behavior and provides targeted marketing strategies for leagues and clubs.

## 2 Theory framework

### 2.1 The theory foundation

The theoretical foundation of this study is relationship marketing theory. Berry (1995) is widely credited with formally introducing the concept of relationship marketing, arguing that firms should not limit their strategic focus to customer acquisition but rather maintain and enhance long-term relationships to foster customer loyalty. Particularly in service contexts, characterized by intangibility and high levels of interaction, continuous interaction and relationship maintenance between firms and customers becomes a vital source of competitive advantage. Since the 1990s, relationship marketing research has shifted from conceptual advocacy to the empirical validation of relational constructs and models.

A central concept within relationship marketing is relationship quality, which refers to the overall strength or closeness of the relationship between a firm and its customers (Vesel and Zabkar, 2010) and is one of the basic frameworks for understanding customer loyalty (De Cannière et al., 2009). Existing studies commonly conceptualize relationship quality as a multidimensional construct comprising satisfaction, trust, and affective commitment (De Cannière et al., 2009; Hennig-Thurau et al., 2002; Liang et al., 2011). Notably, affective commitment and identification exhibit conceptual overlap and are sometimes treated as interchangeable constructs in the literature (Meyer and Allen, 1991; Mowday et al., 1979; Zhou and Bao, 2005). Therefore, this study adopts satisfaction, trust, and team identification as the relational variables to explain the formation of spectator loyalty in the CSL.

### 2.2 The concept of variables

#### 2.2.1 Spectator satisfaction

Consumer satisfaction has been widely conceptualized as an evaluative judgment formed by comparing perceived performance with prior expectations (Anderson et al., 1994). Oliver et al. (1997) define satisfaction as a cognitive–affective evaluation reflecting the degree to which a product or service delivers pleasurable fulfillment in consumption. In summary, satisfaction represents a subjective post-consumption assessment that integrates individuals’ experiences with evaluative standards. In sport events, spectator satisfaction refers to spectators’ pleasurable and favorable responses to the competition value of a sporting event and/or to the ancillary services provided during the event (Van Leeuwen et al., 2002; Yoshida and James, 2010). Game satisfaction captures spectators’ overall evaluation of the core product, namely the match, whereas service satisfaction reflects their overall assessment of the ancillary services offered at the match.

#### 2.2.2 Spectator trust

Trust has been characterized as a willingness to rely on an exchange partner in whom one has confidence (Moorman et al., 1993). Morgan and Hunt (1994) argue that trust is grounded in beliefs about a partner’s reliability and integrity, and that it reduces opportunistic behaviors and conflict within relational exchanges. Consumer trust may be directed toward both organizations and individuals. Trust in organizations refers to confidence in the quality and reliability of the products or services the organization provides (Garbarino and Johnson, 1999). In sport settings, sport consumers often develop trust toward sport organizations, teams, and management structures in ways analogous to interpersonal trust (Kim and Trail, 2011). In this sense, spectator trust reflects beliefs that teams and leagues will operate professionally, uphold fairness, and provide consistent and credible sport consumption experiences.

#### 2.2.3 Team identification

Janssen and Huang (2008) pointed out that team identification refers to the process by which individuals perceive themselves based on the values, goals, attitudes, and behaviors they share with other team members. In sport, team identification is defined as the level of psychological connection an individual feels with a sports entity or team (Kim and Kim, 2009), it emerges when individuals develop the cognition that they support and follow a particular team (Wann et al., 2001). Overall, team identification reflects a deep psychological and emotional attachment that links an individual to a specific sport team. This attachment becomes part of the individual’s self-concept, such that the team’s successes and failures are experienced as one’s own and contribute to a sense of belonging and shared identity with fellow fans.

#### 2.2.4 Spectator loyalty

In sport consumer research, spectator loyalty is divided into attitudinal loyalty and behavioral loyalty. Attitudinal loyalty refers to consumers’ psychological commitment to a product, brand, marketer, or service (Szmigin and Carrigan, 2001). It reflects a generalized attachment and a long-term desire to maintain the relationship. Behavioral loyalty refers to consumers’ (future) behaviors or behavioral intentions (Avourdiadou and Theodorakis, 2014; Howat and Assaker, 2013). In a sport context, behavioral loyalty includes renewing or extending club memberships, increasing membership scope, recommending the club to others, and other repeat-consumption behaviors. The present study focuses on spectator behavioral loyalty, as it represents a direct and observable indicator of relationship outcomes.

### 2.3 The hypothesis

In the sport literature, satisfaction, trust, and team identification are recognized as having positive effects on loyalty. Kuenzel and Yassim (2007) reported that satisfaction directly affected behavioral outcomes such as word-of-mouth and revisit intentions among cricket spectators. Koo et al. (2008) found that satisfaction significantly influenced behavioral intentions among women’s college basketball fans. Research by Lee and Kang’s (2015) in the Korean professional basketball league demonstrated that game-related satisfaction positively influenced both team identification and revisit intentions. In addition, Akoglu and Özbek (2022) indicated that perceived trust among sports consumers significantly predicts their purchase intentions. Kwon et al. (2022), Lee, M. A. et al. (2020), Lee, H. W. et al. (2020), and Matsuoka et al. (2003) consistently found that team identification significantly predicted spectators’ intentions to attend future games.

Existing research has highlighted the mediating roles of trust and team identification. Huang and Kim (2023) demonstrated that trust mediated the relationship between satisfaction and loyalty in fitness center customers. Wu et al. (2012) showed that team identification (at the fan–team level) was a primary determinant of revisit intentions, and trust in the team was a key driver. Bodet and Bernache (2011), studying spectators of a French elite hockey club, found that transaction-specific satisfaction strongly predicted attitudinal loyalty and that team identification mediated the relationships among satisfaction, game attendance, and attitudinal loyalty. Lee and Kang (2015) further found that team identification partially mediated the effect of core game satisfaction on revisit intentions. Argan et al. (2019) demonstrated that team-based trust was significantly associated with team identification, which in turn mediated the relationship between fan trust and behavioral loyalty in the Turkish Super League.

**Figure 1:**
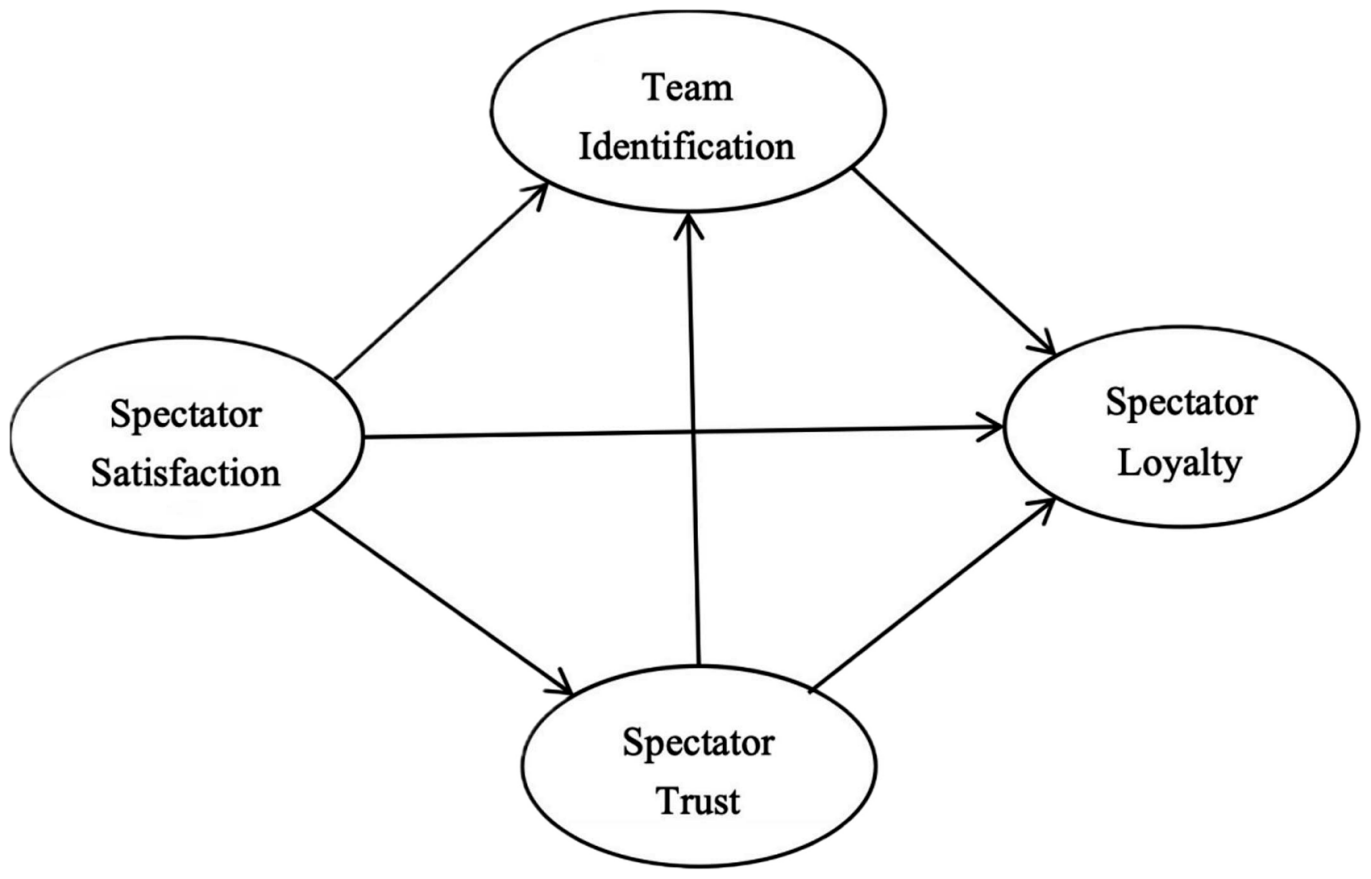
The theoretical model 1.

Based on the above literature, this study proposes the theoretical model 1 (see Figure 1) and following hypothesis and following hypotheses:

M_1_H1: Spectator satisfaction has a significant direct effect on loyalty.

M_1_H2: Spectator trust has a significant direct effect on loyalty.

M_1_H3: Team identification has a significant direct effect on loyalty.

M_1_H4: Spectator satisfaction has a significant direct effect on trust.

M_1_H5: Spectator satisfaction has a significant direct effect on team identification.

M_1_H6: Spectator trust has a significant direct effect on team identification.

M_1_H7: Spectator trust mediates the relationship between spectator satisfaction and loyalty.

M_1_H8: Team identification mediates the relationship between spectator satisfaction and loyalty.

M_1_H9: Spectator satisfaction positively influences loyalty through a sequential mediating mechanism of trust and team identification.

**Figure 2:**
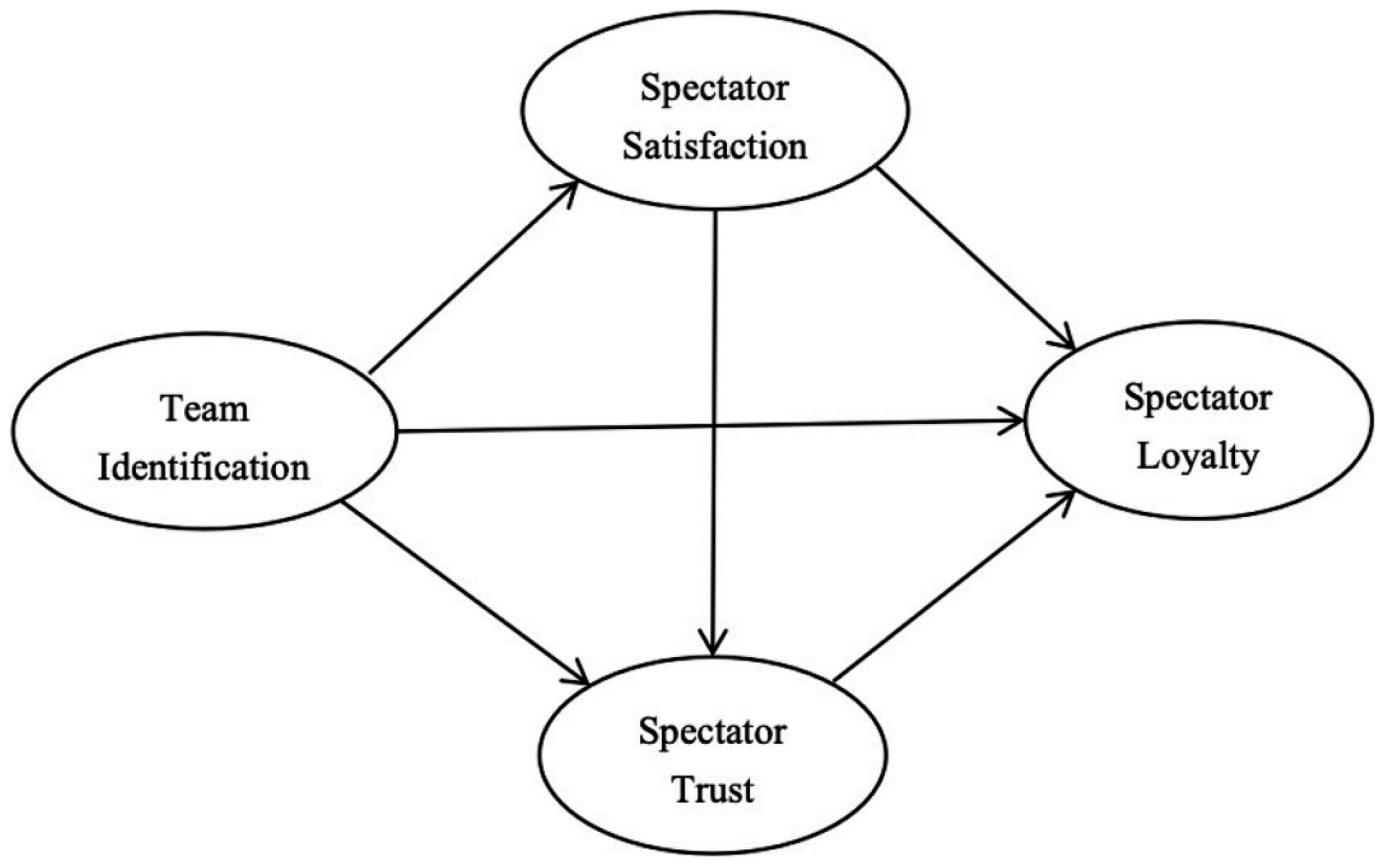
The theoretical model 2.

However, other studies have suggested a different causal direction. Team identification was found to directly strengthen both satisfaction and trust (Pan and Phua, 2021; Suh et al., 2013; Trkulja et al., 2023; Yang and Qin, 2025). Some scholars have argued that satisfaction may act as a mediating variable between team identification and loyalty (Son et al., 2023). Accordingly, this study proposes an alternative theoretical model 2 (see Figure 2) and presents the following hypotheses.

M_2_H1: Team identification has a significant direct effect on loyalty.

M_2_H2: Spectator satisfaction has a significant direct effect on loyalty.

M_2_H3: Spectator trust has a significant direct effect on loyalty.

M^2^H4: Team identification has a significant direct effect on spectator satisfaction.

M_2_H5: Team identification has a significant direct effect on spectator trust.

M_2_H6: Spectator satisfaction has a significant direct effect on trust.

M_2_H7: Spectator satisfaction mediates the relationship between team identification and loyalty.

M_2_H8: Spectator trust mediates the relationship between team identification and loyalty.

M_2_H9: Team identification positively influences loyalty through a sequential mediating mechanism of satisfaction and trust.

## 3 Methods

### 3.1 Instrument development

Based on the conceptual definitions and prior literature, this study developed four scales: spectator satisfaction, spectator trust, team identification, and spectator loyalty. Scale items were adapted from established studies and refined to fit the context of the CSL. To ensure content validity, six football experts evaluated the item relevance and clarity. The expert panel included one national football referee, two CSL match supervisors, and three scholars specializing in professional football. All experts reached consensus on the appropriateness of the items.

A pilot survey was conducted in June 2025 among football-major students at a sports university and members of a football fan community. Eligibility required prior in-person attendance at a CSL match. A total of 142 responses were collected, with 87 valid questionnaires retained after removing incomplete or invalid responses. Two statistical procedures were used to determine whether individual items should be retained: (1) Item Analysis, differences between high- and low-scoring groups were examined for each item. Items that did not show significant differences at *p* < 0.01 were considered for deletion. (2) Correlation Analysis, the item–total correlation for each item was required to exceed 0.40, indicating adequate construct relatedness (Wu, 2010). All items across the four scales demonstrated significant group differences (*p* < 0.01) and item–total correlations above 0.40. Therefore, all items were retained for the formal survey (Table 1).

**Table 1:** Scale items and its literature sources.

Latent variables	Items	Literature sources
Spectator satisfaction	My decision to attend this match was the right one	Sarstedt et al. (2014); Yoshida and James (2010); Van Leeuwen et al. (2002)
	The live match met my spectating needs	
	Overall, I am satisfied with the stadium services	
	The stadium services met my needs	
Spectator Trust	The club prioritizes improving match quality	Kim and Jeong (2025); Wu et al. (2012)
	The club considers fans’ interests when making major decisions	
	The club can be trusted to do the right thing	
Team Identification	The long-term success of the club I support is very important to me	Tsigilis et al. (2023); Bodet and Bernache (2011); Argan et al. (2019); Theodorakis et al. (2010)
	I feel a strong sense of belonging to the club I support	
	When the team I support performs poorly, I often feel frustrated	
	I wear or display symbols that represent the club’s identity when watching matches	
	When others criticize the team, it feels like they are criticizing me personally	
Spectator Loyalty	I will continue to support the club even when its performance is poor	Kim and Trail (2011); Byon et al. (2013); Hennig-Thurau et al. (2002); Yoshida and James (2010)
	I consistently follow the club’s developments (e.g., fixtures, transfers)	
	Attending the club’s matches next season is part of my future plans	
	I would recommend attending the club’s matches to others	
	I will continue to purchase the club’s merchandise or memorabilia	

### 3.2 Questionnaire survey

The finalized questionnaire consisted of four scales measured on a 7-point Likert scale (1 = strongly disagree; 7 = strongly agree). Items captured respondents’ evaluations of their CSL match experiences, trust in the clubs, psychological connection to teams, and loyalty-related behaviors. Data were collected during three CSL matches through onsite distribution of the formal questionnaire: June 14, 2025 and July 19, 2025 at Shenzhen Pengcity FC’s home stadium, Shanghai Shenhua and Qingdao Hainiu were visiting teams. August 2, 2025 at Meizhou Hakka FC’s home stadium, Shanghai Port is the visiting team.

A total of 320 questionnaires were distributed, and 278 valid responses were obtained after excluding 42 invalid questionnaires (due to uniform response patterns or missing data), yielding a valid response rate of 86.9%. Among the respondents, 256 were male and 22 were female. The largest group of participants was under 20 years old (*n* = 87), followed by those aged 21–30 (*n* = 80), 31–40 (*n* = 72), 41–50 (*n* = 30), 51–60 (*n* = 8), and over 60 (*n* = 1).

### 3.3 Data analysis

The confirmatory factor analysis (CFA) was conducted by using AMOS 23.0 to evaluate the structural validity of the measurement models. The model fit was assessed using a range of commonly applied indices, including the Chi-square to degrees of freedom ratio (*χ*^2^/df), Root Mean Square Error of Approximation (RMSEA), Goodness-of-Fit Index (GFI), Adjusted Goodness-of-Fit Index (AGFI), Normed Fit Index (NFI), Relative Fit Index (RFI), Incremental Fit Index (IFI), Tucker–Lewis Index (TLI), Comparative Fit Index (CFI), Parsimonious Normed Fit Index (PNFI), and Parsimonious Goodness-of-Fit Index (PGFI). In addition, the study used the SPSS PROCESS macro to test the indirect effects. Bootstrapping with 5,000 resamples was used to generate bias-corrected confidence intervals, ensuring rigorous estimation of both simple and chain mediation effects.

## 4 Results

Table 2 presents the goodness-of-fit indices for Model 1 and Model 2. The fit statistics of the two models are identical, indicating that they are equivalent models. The *χ*^2^/df, RMSEA, PNFI, and PGFI values all met the recommended thresholds. The GFI value approached 0.90. AGFI, NFI and RFI did not reach the suggested standard; however, these indicators are all highly sensitive to sample size. IFI, TLI, and CFI are relatively insensitive to sample size, CFI shows robust performance even in smaller samples (Marsh et al., 1988). The IFI, TLI, and CFI all exceeded 0.90, indicating that Model 1 and Model 2 both achieved a satisfactory overall fit.

**Table 2:** Results of structural model fit assessment.

Indicators		Recommended Criteria	Model 1	Model 2
Absolute fit indices	*χ*^2^/df	<3.0	2.579	2.579
	RMSEA	<0.1	0.076	0.076
	GFI	>0.9	0.889	0.889
	AGFI		0.846	0.846
Incremental fit indices	NFI	>0.9	0.883	0.883
	RFI		0.857	0.857
	IFI		0.925	0.925
	TLI		0.907	0.907
	CFI		0.924	0.924
Parsimonious fit indices	PNFI	>0.5	0.721	0.721
	PGFI		0.645	0.645

**Figure 3:**
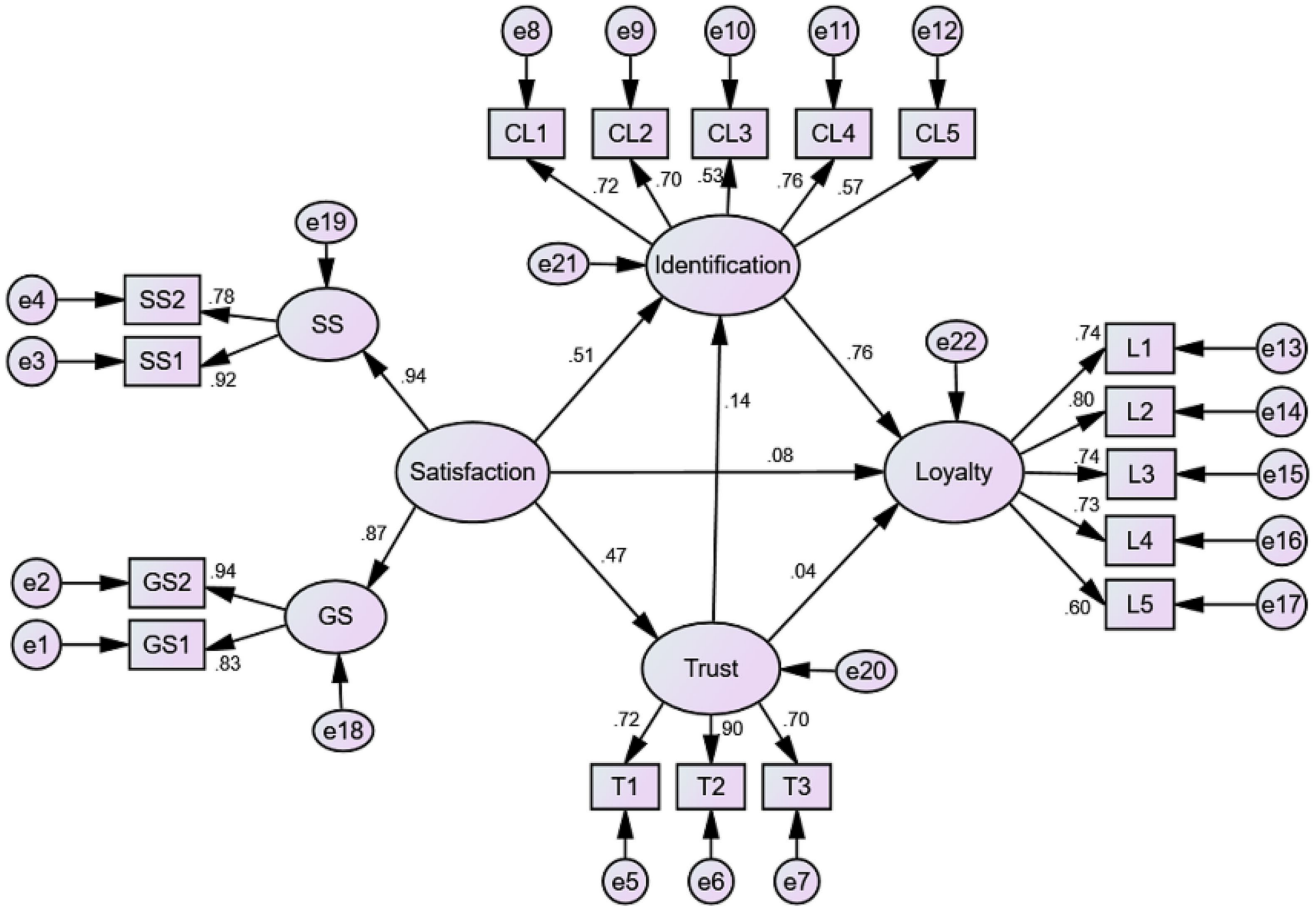
Structural path coefficients of Model 1.

**Figure 4:**
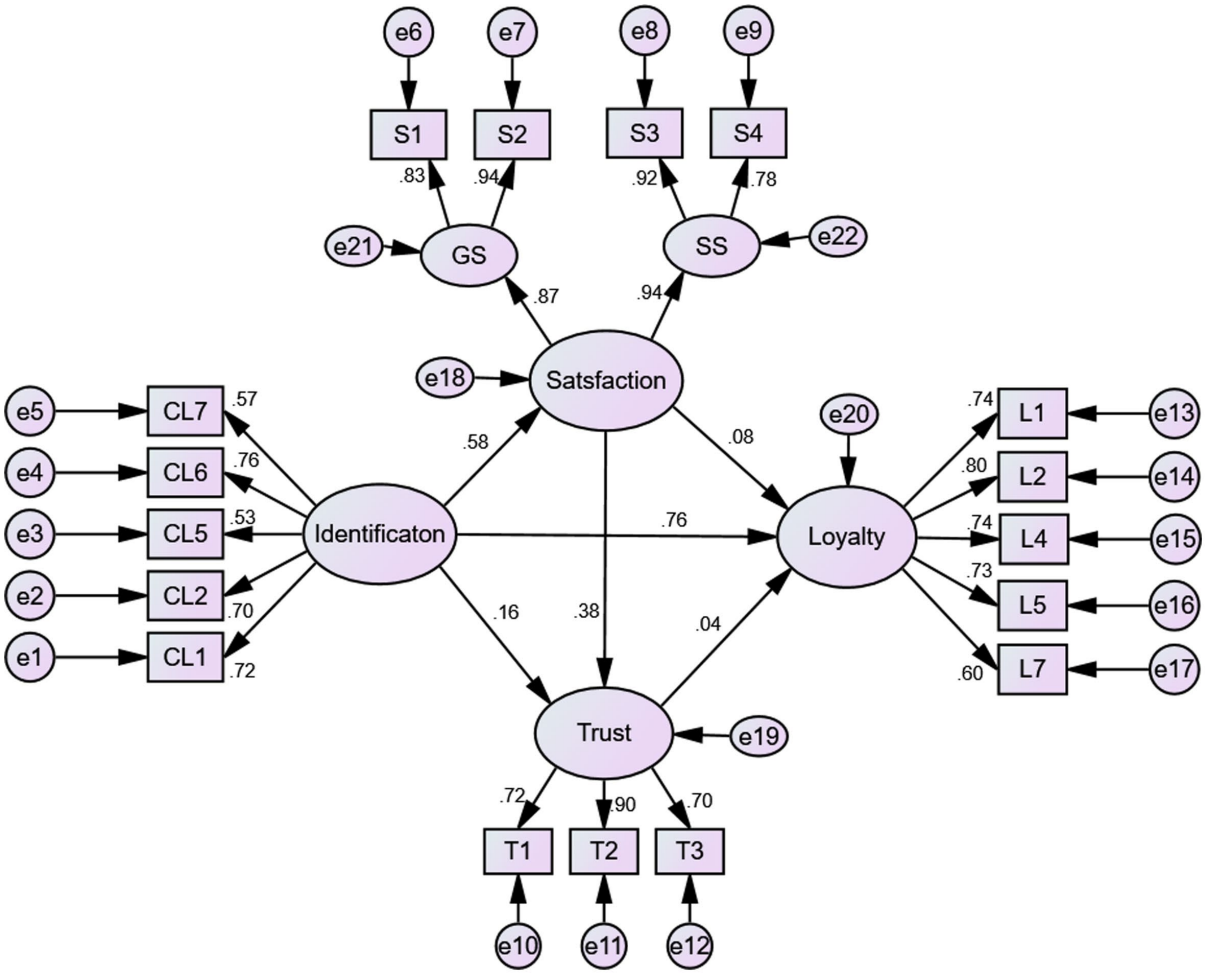
Structural path coefficients of Model 2.

Figures 3, 4, along with Table 3, display the path coefficients and their significance in both models. In Model 1, hypotheses M_1_H1 and M_1_H2 were rejected, indicating that spectator satisfaction and trust have no significant direct effects on loyalty; M_1_H3, M_1_H4, M_1_H5, and M_1_H6 were supported, indicating that spectator satisfaction significantly predicts trust and team identification, team identification significantly predicts loyalty, and trust has a weak but meaningful direct effect on team identification (marginally significant at *p* < 0.10). In addition, satisfaction was divided into game satisfaction and service satisfaction. Separate analyses showed no substantive differences in their effects when each dimension was entered independently into the model (see Table 4).

**Table 3:** Path coefficient values of the structural model 1 and model 2.

Path		SE	S. E.	C. R.	P	Hypothesis
Model 1	M_1_H1: Satisfaction→Loyalty	0.076	0.097	1.025	0.305	Reject
	M_1_H2: Trust→Loyalty	0.037	0.066	0.606	0.644	Reject
	M_1_H3: Identification→Loyalty	0.759	0.116	7.978	0.000	Support
	M_1_H4: Satisfaction→Trust	0.470	0.095	6.008	0.000	Support
	M_1_H5: Satisfaction→Identification	0.511	0.093	5.909	0.000	Support
	M_1_H6: Trust→Identification	0.137	0.067	1.829	0.067	Support
Model 2	M_2_H1: Identification→Loyalty	0.759	0.112	7.764	0.000	Support
	M_2_H2: Satisfaction→Loyalty	0.076	0.097	1.025	0.305	Reject
	M_2_H3: Trust→Loyalty	0.037	0.066	0.606	0.544	Reject
	M_2_H4: Identification→Satisfaction	0.576	0.074	6.880	0.000	Support
	M_2_H5: Identification→Trust	0.160	0.094	1.826	0.068	Support
	M_2_H6: Satisfaction→Trust	0.378	0.111	4.128	0.000	Support

**Table 4:** Path coefficients for the two dimensions of satisfaction as independent variables.

Paths		Estimate	S. E.	C. R.	P
Model 1a	GS→Trust	0.390	0.075	5.369	0.000
	Trust→Identification	0.198	0.064	2.745	0.006
	GS→Identification	0.451	0.071	5.782	0.000
	Trust→Loyalty	0.049	0.064	0.825	0.409
	Identification→Loyalty	0.772	0.114	8.232	0.000
	GS→Loyalty	0.049	0.074	0.734	0.463
Model 1b	SS→Trust	0.465	0.088	6.151	0.000
	Trust→Identification	0.162	0.068	2.124	0.034
	SS→Identification	0.462	0.086	5.579	0.000
	Trust→Loyalty	0.038	0.066	0.614	0.539
	Identification→Loyalty	0.767	0.113	8.198	0.000
	SS→Loyalty	0.069	0.089	0.973	0.330

Model 1a and Model 1b indicate that game satisfaction and service satisfaction were included as independent variables in the models, respectively; GS is game satisfaction; SS is service satisfaction.

In Model 2, hypotheses M_2_H2 and M_2_H3 were rejected, indicating that spectator satisfaction and trust have no significant direct effects on loyalty. Hypotheses M_2_H1, M_2_H4, M_2_H5, and M_2_H6 were supported. These findings indicate that team identification has significant direct effects on both spectator satisfaction and loyalty, team identification has a weak but meaningful direct effect on trust (marginally significant at *p* < 0.10), and satisfaction has a significant direct effect on trust.

Table 5 shows the results of the mediation analyses for Model 1 and Model 2. A 95% bootstrap confidence interval that includes zero indicates that the estimated indirect effect is not statistically significant at the 5% alpha level. In Model 1, M_1_H7 was rejected, while M_1_H8 and M_1_H9 were supported. Specifically, trust did not significantly mediate the relationship between satisfaction and loyalty (effect size = 0.033), team identification significantly mediated this relationship (effect size = 0.242), trust and team identification jointly produced a significant chain-mediating effect between satisfaction and loyalty (effect size = 0.034). In Model 2, M_2_H7 was supported, M_2_H8 and M_2_H9 were rejected. In this model, satisfaction significantly mediated the relationship between team identification and loyalty (effect size = 0.071). By contrast, the mediating effect of trust between team identification and loyalty was not significant (effect size = 0.012), and the chain-mediating effect of satisfaction and trust was also not statistically significant (effect size = 0.013).

**Table 5:** Test of mediating effects in model 1 and model 2.

Paths		Effect size	Boot SE	Boot LLCI	Boot ULCI	Hypothesis
Model 1	Total mediating effect	0.309	0.043	0.226	0.396	–
	M_1_H7: (Ind1) Satisfaction→Trust→Loyalty	0.033	0.027	−0.019	0.087	Reject
	M_1_H8: (Ind2) Satisfaction→Identification→Loyalty	0.242	0.039	0.167	0.320	Support
	M_1_H9: (Ind3) Satisfaction→Trust→Identification→Loyalty	0.034	0.016	0.007	0.070	Support
	C1 (ind1 minus ind2)	−0.210	0.051	−0.307	−0.113	–
	C2 (ind1 minus ind3)	−0.001	0.034	−0.071	0.062	–
	C3 (ind2 minus ind3)	0.209	0.044	0.120	0.292	–
Model 2	Total mediating effect	0.097	0.030	0.037	0.156	–
	M_2_H7: (Ind1) Identification→Satisfaction→Loyalty	0.071	0.029	0.016	0.129	Support
	M_2_H8: (Ind2) Identification→Trust→Loyalty	0.012	0.011	−0.010	0.036	Reject
	M_2_H9: (Ind3) Identification→Satisfaction→Trust→Loyalty	0.013	0.011	−0.008	0.037	Reject
	C1 (ind1 minus ind2)	0.059	0.034	−0.005	0.128	–
	C2 (ind1 minus ind3)	0.058	0.034	−0.008	0.126	–
	C3 (ind2 minus ind3)	0.000	0.008	−0.022	0.014	–

## 5 Discussion

### 5.1 The main finding of the study

#### 5.1.1 Direct effects of satisfaction, trust and team identification on loyalty

This study found that team identification exerted a significant direct effect on spectator loyalty in both Model 1 and Model 2. This finding is consistent with the meta-analysis conducted by Kwon et al., which reported a medium-to-large effect size between team identification and sport consumer loyalty. Team identification is theoretically grounded in Social Identity Theory, which posits that individuals define their self-concept through membership in social groups to achieve a sense of belonging and meaningful social identity (Tajfel, 2010). Spectators are not merely rational consumers but social actors who derive part of their self-concept from group memberships. Sport teams function as salient social categories capable of providing symbolic meaning, emotional resonance, and a sense of belonging (Ashforth and Mael, 1986; Fink et al., 2002). Through this sense of “we-ness,” fans tend to demonstrate greater supportive behaviors toward the club—such as repeatedly attending matches and purchasing club merchandise—as a way of expressing and reinforcing their social identity (Hirshon, 2020).

The prevailing view is that spectator satisfaction and trust exert a direct positive effect on loyalty (Loranca-Valle et al., 2021). However, this study found that neither satisfaction nor trust had a significant direct effect on loyalty in both Model 1 and 2. One possible explanation is that the formation of loyalty requires time for clubs to become embedded within local identity structures and collective memory. Established in 1994, the CSL has a relatively short history, resulting in limited brand heritage and insufficient football culture. Furthermore, frequent club renaming, ownership withdrawal, and team dissolution in the CSL have disrupted the continuity of loyalty targets and weakened stable psychological anchors. When organizational persistence is lacking, satisfaction and trust are more likely to remain transactional evaluations rather than evolve into enduring commitments. Therefore, loyal fans represent a relatively smaller segment in the current CSL spectator base (Jia et al., 2020).

#### 5.1.2 The mediating role of satisfaction and team identification

This study found that spectator satisfaction had a significant direct effect on team identification, and team identification played a significant mediating role between spectator satisfaction and loyalty in Model 1. This aligns with prior evidence from French ice hockey (Bodet and Bernache, 2011), French football (Huiszoon, 2019), and Korean professional basketball (Lee and Kang, 2015), all of which emphasize that satisfaction alone is insufficient to sustain loyalty without the presence of a strong psychological bond with the team. Moreover, this study found that team identification had a significant direct effect on spectator satisfaction, and satisfaction significantly mediated the relationship between team identification and spectator loyalty in Model 2. This finding is consistent with the results reported by Son et al. (2023) in the Korean professional baseball league. Prior studies have found that spectators with higher levels of team identification are more likely to experience satisfaction (Gau et al., 2007; Wann and Branscombe, 1993). Spectators who exhibit both high team identification and high satisfaction are more likely to participate in future games (Matsuoka et al., 2003).

Although satisfaction and team identification exhibit a reciprocal relationship, the mediating effect of team identification in the satisfaction—loyalty pathway (0.242) is substantially stronger than that of satisfaction in the identification—loyalty pathway (0.071). This finding highlights a distinction in their roles within the loyalty formation process among CSL spectators. Satisfaction appears to serve as an initial catalyst for the development of team identification. Once team identification is established, it further enhances satisfaction, thereby creating a reciprocal dynamic that jointly fosters loyalty.

#### 5.1.3 Chain mediation of trust and identification

This study found that trust and team identification exerted marginally significant effects on each other. Prior research likewise suggests a mutual relationship between these two constructs. Fans’ trust in their team significantly enhances team identification (Wu et al., 2012), whereas highly identified fans are more likely to exhibit stronger brand trust regardless of team performance or game location (Pan and Phua, 2021). However, their mediating effects differed in this study. Trust did not significantly mediate the relationship between team identification and loyalty, nor did satisfaction and trust jointly mediate the identification—loyalty pathway. In contrast, trust and team identification exerted a significant chain mediation effect in the satisfaction—loyalty pathway. This finding is consistent with the results of Argan et al. (2019) in the Turkish Super League.

From a relationship marketing perspective, satisfaction is widely recognized as a key antecedent of trust, as positive experiences reinforce perceptions of reliability, competence, and integrity (Garbarino and Johnson, 1999; Morgan and Hunt, 1994). Kramer (1993) argues that team identification develops alongside group-based trust, as trust reduces perceived interpersonal and organizational risks, thereby facilitating commitment to the collective. Related evidence from organizational psychology further suggests that trust promotes identification by encouraging individuals to prioritize collective goals over personal concerns (Han and Harms, 2010).

Compared with mature professional leagues in Europe and North America, the CSL remains in a phase of institutional transition and market restructuring. In recent years, it has experienced investment volatility, frequent club renaming, wage arrears, and other destabilizing events. The quality of matches and related services has fluctuated considerably, and the league has periodically faced crises of trust. Under such highly uncertain conditions, satisfaction with the game and peripheral services is a crucial psychological safeguard for spectators’ trust. Trust reduces perceived risk and strengthens spectators’ confidence in the club’s long-term development. When spectators perceive the club as demonstrating integrity, professionalism, and stable governance, they are more willing to psychologically align themselves with the team and incorporate it into their social identity, thereby transforming an exchange-based relationship into an identification-based attachment.

### 5.2 Managerial implications

This study’s resulting offer several actionable insights for CSL clubs and league organizers. First, prioritize the cultivation of spectator identification. Spectator loyalty in the CSL is primarily driven by team identification rather than direct satisfaction or trust. Clubs should focus on cultivating long-term attachment through coherent team narratives, promotion of local cultural symbols, fan engagement programs, and initiatives that reinforce a sense of community belonging. Second, enhance game quality and match day experience. High-quality sporting competition—characterized by competitive intensity, consistent performance, and a distinctive team image—can facilitate the internalization of the team as part of spectators’ self-concept. In addition, the stadium should be understood not merely as a physical venue, but as a social arena in which shared symbols, collective rituals, and interactive experiences are enacted. For example, the coordinated display of tifo banners and the collective singing of the club anthem can strengthen emotional attachment and transform episodic satisfaction into a more enduring psychological bond. Third, strengthen organizational credibility. Clubs should emphasize transparent governance, professional management, and consistent communication with supporters. By enhancing organizational credibility, clubs can more effectively convert satisfied spectators into highly identified and loyal fans.

### 5.3 Limitations

This study has two limitations that should be addressed in future research. First, according to the 2019 CSL Commercial Value Report jointly released by Deloitte and the Chinese Football Association, male fans account for 61% of the loyal fan base of the CSL, while female fans represent 39, and 81% of fans are under the age of 40 (Deloitte, 2020). In this study, 86% of the sample was under 40 years old, which is consistent with the official report; however, female respondents accounted for only 8% of the sample. This limits the generalizability of the findings to some extent; future research should include more female samples to explore potential gender differences in the paths of loyalty formation. Second, this study focuses solely on in-stadium experiences and does not account for the rapidly expanding digital engagement in Chinese professional sport. Contemporary fandom increasingly extends beyond physical attendance to include online viewing, social media interaction, and participation in virtual fan communities, which play important roles in identity construction and community building. Future research should capture e-fan loyalty and better reflect the hybrid nature of contemporary sport consumption.

## 6 Conclusion

This study examined the roles and sequential order of satisfaction, trust, and team identification in the formation of spectator loyalty within the CSL. The findings indicate that neither satisfaction nor trust exerts a significant direct effect on loyalty, whereas team identification demonstrates a strong and significant direct influence. Moreover, three indirect pathways were identified: Satisfaction→Team Identification→Loyalty, Team Identification→Satisfaction→Loyalty, and Satisfaction→Trust→Team Identification→Loyalty. The first indirect pathway exhibits the largest effect size.

The theoretical contribution of this study lies in advancing a configurational understanding of loyalty formation in emerging professional football markets. Rather than emerging from isolated linear effects, loyalty is shaped by the dynamic interplay of multiple psychological constructs, team identification serves as a pivotal structural anchor. This study also provides stronger empirical evidence for league authorities and clubs in formulating relationship marketing strategic priorities.

## Data Availability

The original contributions presented in the study are included in the article/supplementary material, further inquiries can be directed to the corresponding author.
